# Glutamate dependent NMDA receptor 2D is a novel angiogenic tumour endothelial marker in colorectal cancer

**DOI:** 10.18632/oncotarget.7812

**Published:** 2016-03-01

**Authors:** Henry J.M. Ferguson, Joseph W. Wragg, Stephen Ward, Victoria L. Heath, Tariq Ismail, Roy Bicknell

**Affiliations:** ^1^ Molecular Angiogenesis Group, Institute for Biomedical Research, School of Immunity and Infection, College of Medical and Dental Sciences, University of Birmingham, Edgbaston, Birmingham, B15 2TT, UK; ^2^ Queen Elizabeth Hospital, Queen Elizabeth Medical Centre, Edgbaston, Birmingham, B15 2TH, UK

**Keywords:** colorectal cancer, GRIN2D, tumour endothelial marker, vaccination, active immunotherapy

## Abstract

Current vascular-targeted therapies in colorectal cancer (CRC) have shown limited benefit. The lack of novel, specific treatment in CRC has been hampered by a dearth of specific endothelial markers. Microarray comparison of endothelial gene expression in patient-matched CRC and normal colon identified a panel of putative colorectal tumour endothelial markers. Of these the glutamate dependent NMDA receptor GRIN2D emerged as the most interesting target. GRIN2D expression was shown to be specific to colorectal cancer vessels by RTqPCR and IHC analysis. Its expression was additionally shown be predictive of improved survival in CRC. Targeted knockdown studies *in vitro* demonstrated a role for GRIN2D in endothelial function and angiogenesis. This effect was also shown *in vivo* as vaccination against the extracellular region of GRIN2D resulted in reduced vascularisation in the subcutaneous sponge angiogenesis assay. The utility of immunologically targeting GRIN2D in CRC was demonstrated by the vaccination approach inhibiting murine CRC tumour growth and vascularisation. GRIN2D represents a promising target for the future treatment of CRC.

## INTRODUCTION

Colorectal cancer is the third most common cancer globally, making up about 10% of all malignant diagnoses [1]. There are over 1.4 million new cases annually, 65% of which are found in developed countries, with 695,000 deaths from the disease. Early stage colorectal cancer, confined to the wall of the colon, is usually curable with surgery. More advanced disease, making up 50% of cases in the UK, has a poorer prognosis, and often requires the addition of adjuvant therapies such as chemotherapy [1].

The five-year survival rate in developed countries is around 65%, however, this drops to below 10% with metastatic disease [2]. Curative treatment heavily relies upon the achievement of a histologically clear resection margin at surgery [3, 4] and although 80% of CRC resections do achieve this, 50% will relapse to metastatic disease, due to the presence of micro-metastases present at the time of resection [2, 5]. Improvements in treatment and prevention of metastatic disease, are of vital importance to achieve better outcomes in CRC.

Adjuvant therapeutic agents have been in established use in the treatment of colorectal cancer (CRC) for over 50 years [6], but despite introduction of new chemotherapeutic agents, and the introduction of monoclonal antibodies, presentation with macroscopic or microscopic metastatic disease still carries a poor prognosis [7]. This is partly because primary colorectal cancer remains a difficult target for immune therapies [8], with no currently licenced immunotherapies for the Dukes A-C disease. Cetuximab, bevacizumab, and a third monoclonal antibody, panitumumab, are also currently licenced for use in the treatment of advanced CRC, although evidence of benefit is limited, with response rates below 5% when used as a monotherapy [9]. Targeting of the vasculature of CRC has been shown to be a mechanism of interest for novel therapies [10], with anti-angiogenic agents already licensed for advanced disease. However, vascular targeting in the form of a targeted specific immunotherapy, or vaccination, in CRC has not been previously described.

The development of antigen-specific immunotherapies relies upon identification of a specific, over-expressed antigen in the malignancy of interest [11]. Previous work from our group has identified both ROBO4 [12] and CLEC14A [13] as endothelial specific targets in human malignancy. The breaking of immune self tolerance to ROBO4, through vaccination, has been shown to result in decreased tumour growth through anti-angiogenic effects in a subcutaneous murine Lewis Lung Carcinoma model [14].

This article describes the identification of GRIN2D (also known as NMDAR2D, NR2D, GluN2D), a subunit of a glutamate dependent, ionotropic NMDA receptor, and facilitator of cellular calcium influx, previously found in neuronal tissues, as a putative vascular endothelial target in CRC.

## RESULTS

In order to identify novel tumour vascular markers in CRC, comparative analysis of microarray data of four endothelial isolates from colorectal cancer and patient matched healthy colon tissue was conducted. The generation of the endothelial isolates and raw microarray data is described in Wragg *et al*., 2015 [15].

A number of known tumour associated angiogenic genes were identified by this analysis (Table 1), including interleukin 8, angiopoietin 2 and lysyl oxidase like 2, validating the approach as a method for identifying genes enriched on tumour endothelium. This analysis additionally identified a host of collagens (Supplementary Table S1) and matrix metallopeptidases (Supplementary Table S2), key components of active extracellular matrix remodelling, important for endothelial migration and angiogenesis within the tissue. This data collectively implies a pattern of active angiogenesis occurring within the tumour, outstripping that of the surrounding healthy colon tissue.

**Table 1:** Transmembrane genes greater than 5 fold enriched in colorectal cancer vessels

Gene ID	Gene Symbol	GeneBank accession no.	LogFC	P-value
EGF-like-domain, multiple 6	EGFL6	NM_001167890	4.86	0.00
thrombospondin 2	THBS2	NM_003247	4.26	0.00
fibroblast activation protein, alpha	FAP	NM_004460	4.11	0.00
leucine zipper, putative tumor suppressor 1	LZTS1	NM_021020	4.03	0.00
Thy-1 cell surface antigen	THY1	NM_006288	3.87	0.00
Fc fragment of IgG, low affinity IIIa, receptor (CD16a)	FCGR3A	NM_000569	3.58	0.00
interleukin 6 (interferon, beta 2)	IL6	NM_000600	3.40	0.00
stimulated by retinoic acid gene 6 homolog (mouse)	STRA6	NM_001199042	3.15	0.00
plexin domain containing 1	PLXDC1	NM_020405	3.12	0.01
potassium inwardly-rectifying channel, subfamily J, member 8	KCNJ8	NM_004982	2.97	0.00
placental growth factor	PGF	NM_002632	2.92	0.01
pyrimidinergic receptor P2Y, G-protein coupled, 6	P2RY6	NM_176798	2.87	0.00
glutamate receptor, ionotropic, N-methyl D-aspartate 2D	GRIN2D	NM_000836	2.83	0.01
platelet-derived growth factor receptor, beta polypeptide	PDGFRB	NM_002609	2.82	0.00
frizzled family receptor 10	FZD10	NM_007197	2.81	0.00
tribbles homolog 3 (Drosophila)	TRIB3	NM_021158	2.81	0.01
regulator of G-protein signaling 5	RGS5	NM_003617	2.79	0.00
claudin 2	CLDN2	NM_001171092	2.75	0.02
wingless-type MMTV integration site family member 2	WNT2	NM_003391	2.75	0.00
uncharacterized LOC541471	LOC541471	NR_015395	2.73	0.01
leukocyte immunoglobulin-like receptor, subfamily B, member 4	LILRB4	BC026309	2.71	0.01
solute carrier family 4, sodium borate transporter, member 11	SLC4A11	NM_032034	2.67	0.01
macrophage receptor with collagenous structure	MARCO	NM_006770	2.67	0.01
vasorin	VASN	NM_138440	2.66	0.00
transglutaminase 2	TGM2	NM_198951	2.60	0.00
sulfatase 1	SULF1	NM_015170	2.58	0.02
leucine rich repeat and Ig domain containing 1	LINGO1	NM_032808	2.57	0.00
SLAM family member 8	SLAMF8	NM_020125	2.52	0.03
olfactory receptor, family 51, subfamily E, member 1	OR51E1	NM_152430	2.50	0.00
gap junction protein, alpha 4, 37kDa	GJA4	NM_002060	2.50	0.00
interleukin 13 receptor, alpha 2	IL13RA2	NM_000640	2.45	0.02
regulator of G-protein signaling 16	RGS16	NM_002928	2.44	0.01
fibronectin type III domain containing 1	FNDC1	NM_032532	2.39	0.02
notch 3	NOTCH3	NM_000435	2.34	0.00
ArfGAP with dual PH domains 2	ADAP2	NM_018404	2.87	0.00

### Selection of genes of interest

In order to identify potential endothelial markers in colorectal cancer, which can then be used to target therapeutics to the tumour vascular bed, a short list of genes of interest was generated (Table 2). This list detailed genes at least 5 times enriched on colorectal cancer vessels, with a p-value <0.05 and with considerable evidence of trans-plasma-membrane expression. This was done to ensure that selected genes had both considerable differential expression, but were also directly targetable from the bloodstream. This would simplify the mode of delivery of any potential therapeutics targeted against them. A literature review was undertaken on all targets, and those with prior published evidence in the context of colorectal cancer endothelium were excluded from further study. This left eight candidate targets: stimulated by retinoic acid gene 6 (STRA6); glutamate receptor, ionotropic, N-methyl D-aspartate 2D (GRIN2D); fibronectin type III domain containing 1 (FNDC1); G protein-coupled receptor 4 (GPR4); regulator of G-protein signalling 5 (RGS5); transglutaminase 2 (TGM2); potassium inwardly-rectifying channel, subfamily J, member 8 (KCNJ8) and olfactory receptor, family 51, subfamily E, member 1 (OR51E1).

**Table 2:** Known tumour associated or angiogenic genes enriched in colorectal cancer vessels

Gene ID	Gene Symbol	GeneBank accession no.	LogFC	P-value
interleukin 8	IL8	NM_000584	3.42	0.00
angiopoietin 2	ANGPT2	NM_001147	3.40	0.00
plexin domain containing 1	PLXDC1	NM_020405	3.12	0.01
lysyl oxidase-like 2	LOXL2	NM_002318	3.11	0.01
hairy/enhancer-of-split related with YRPW motif-like	HEYL	NM_014571	3.01	0.00
placental growth factor	PGF	NM_002632	2.92	0.01
platelet-derived growth factor receptor, beta polypeptide	PDGFRB	NM_002609	2.82	0.00
endothelial cell-specific molecule 1	ESM1	NM_007036	2.63	0.00
CD86 molecule	CD86	NM_006889	2.42	0.05
apelin	APLN	NM_017413	1.98	0.00
insulin-like growth factor binding protein 7	IGFBP7	NM_001553	1.93	0.01
angiopoietin-like 2	ANGPTL2	NM_012098	1.65	0.09
major histocompatibility complex, class II, DR alpha	HLA-DRA	NM_019111	1.65	0.05
tenascin C	TNC	NM_002160	1.42	0.12
secreted protein, acidic, cysteine-rich (osteonectin)	SPARC	NM_003118	1.39	0.13
neuropilin 1	NRP1	NM_001024629	1.23	0.08
lysyl oxidase	LOX	NM_002317	1.20	0.21
anthrax toxin receptor 1	ANTXR1	NM_053034	1.01	0.20

### Validation of genes of interest

**Figure 1:**
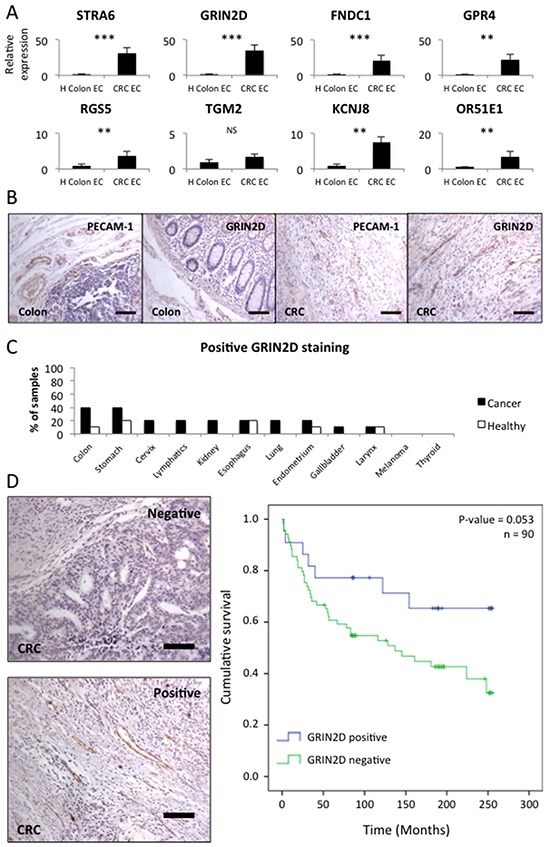
GRIN2D is a specific marker of tumour vasculature in colorectal cancer **A.** quantitative real-time analysis of relative candidate target gene levels in endothelium isolated from malignant and healthy tissue. Gene expression levels were normalised to flotillin-2. Average gene expression ± SEM (n=8, Mann-Whitney U, ***p<0.0001, **p<0.001). **B.** representative images of PECAM-1 and GRIN2D staining in healthy colon and colorectal cancer (scale bar = 50 μm). **C.** multi-organ tissue array analysis of GRIN2D expression, showing the frequency of positive GRIN2D vascular staining. **D.** representative images of positive and negative GRIN2D staining in CRC by IHC (scale bar = 50 μm) and Kaplan-Meier analysis of survival in CRC with (blue) or without (green) GRIN2D vascular expression. Statistical analysis = Log-ranks test, P and N-numbers shown.

RTqPCR was conducted on the eight selected candidate targets, in order to compare gene expression in colorectal cancer endothelial cells (EC) and healthy colon EC (n=8 for each) (Figure 1A). Gene expression was normalised to both flotillin-2 (per cell expression level) and PECAM-1 (per endothelial cell expression level) in order to assess the level of enrichment of the target in the cancer endothelium (Table 3). Additionally, target gene expression level in the cancer EC was compared to flotillin-2 expression, in order to ensure that genes were expressed at a high enough level to be of interest (Table 3). This investigation identified GRIN2D alone as having sufficient cancer enrichment and expression, to be taken forward for further investigation.

**Table 3:** Selection of genes of interest for further investigation and validation based on gene enrichment and expression level in the tumour endothelial isolates

	Flotillin-2 vs. target fold expression change	PECAM vs. target fold expression change	Expression level relative to flotillin-2 (%)	Selected genes
STRA6	30.7	5.1	10.6	
GRIN2D	35.4	15.8	35.0	GRIN2D
FNDC1	20.5	2.4	16.9	
GPR4	22.7	8.8	17.3	
RGS5	3.7	1.0	109.1	
TGM2	1.7	0.5	841.7	
KCNJ8	7.5	6.25	4.4	
OR51E1	7.1	4.0	8.8	

Differential expression of GRIN2D was further validated on the protein level by immunohistochemistry (IHC) on formalin fixed paraffin embedded sections (Figure 1B). Sections of colorectal cancer and healthy colon were stained for PECAM-1 (a marker of endothelium) and GRIN2D by IHC. This analysis determined that the vessels in colorectal cancer stain strongly for GRIN2D, however, vessels in the healthy colon, marked by PECAM-1 staining do not stain for GRIN2D (Figure 1B). Additionally GRIN2D IHC staining was assessed by multi-organ tissue array analysis, in order to determine its global tumour and healthy tissue expression profile (Figure 1C). This involved staining 10 samples each of 12 different tumour and matched healthy tissue sample for GRIN2D. The analysis identified GRIN2D to be positively expressed on the vessels of 40% of colorectal cancers, but only 10% of healthy colon samples. It was additionally expressed on a range of cancer and healthy tissues to a lesser extent. The fact that GRIN2D is expressed to a greater extent in cancerous tissues and in colon cancer in particular, is encouraging in that it suggests a potential for vessel specific targeting, but normal tissue expression would necessitate careful monitoring for tissue toxicity.

In order to investigate whether the expression of GRIN2D has any prognostic value in colorectal cancer, 90 colorectal tumours were stained for GRIN2D (Figure 1D). Key clinical data for this cohort is shown in Supplementary Table S3. The survival of patients with tumours positive or negative for GRIN2D staining was compared by log-ranks statistical analysis (Figure 1D). This analysis determined that patients with positive vascular GRIN2D expression survived for longer than those without, though not to a significant level (p=0.053).

### *In vitro* effects of GRIN2D knockdown on angiogenesis

In order to investigate the functional role GRIN2D plays in tumour endothelium, human umbilical cord vein endothelial cells (HUVEC) were transfected with small interfering RNA (siRNA) duplexes corresponding to GRIN2D, to selectively silence its expression. Gene silencing and protein depletion was confirmed by RTqPCR and western blot analysis (Supplementary Figure S1). This revealed a 60-70% reduction in GRIN2D expression in the presence of GRIN2D siRNA duplexes.

**Figure 2:**
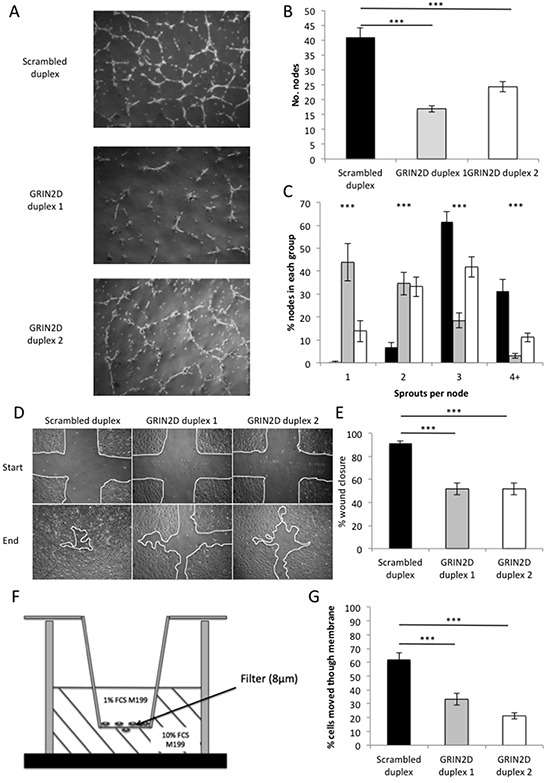
Loss of GRIN2D impairs endothelial function in *in vitro* angiogenesis assays GRIN2D was knocked down by transfection of two siRNA duplexes into 3 separate HUVEC isolates. **A–C**, the cells were plated on matrigel and endothelial tube formation and integrity observed over 16 hours. A, representative images of tube formation in each condition. B, the average number of nodes per field of view ± SEM. C, the average number of sprouts per node ± SEM (n=6 per condition [3] and isolate [3]). **D–E.** HUVEC were plated and allowed to grow to confluence. The monolayer was scratched and wound closure observed at nine scratch intersections over time. D, representative images of wound closure from initial scratch to end-stage. E, quantification of percentage wound closure over the time course of the experiment ± SEM (n=9 per condition [3] and isolate [3]). **F–G.** HUVEC were subjected to the transfilter (modified Boyden chamber) assay, the set up of which is shown in F. G, quantification of the average percentage of endothelial cells that have migrated through the filter, after 16 hours, per field of view ± SEM (n=6 per condition [3] and isolate [3]). Statistical analysis for all, Mann-Whitney U, ***p<0.0001.

The cell-to-cell communication required for endothelial cells to differentiate into vascular tubes is vitally important for their healthy function. To assess the impact that the partial loss of GRIN2D expression has on this process, a matrigel tube-forming assay was performed. By assessing the complexity of network endothelial cells are able to form when grown on the matrigel, their functionality can be determined. This is done by quantifying the number of nodes (network intersections) and sprouts per node, in this model of endothelial capillary formation. The capillary networks formed by endothelial cells from three HUVEC cords showed significant reduction in both the number of nodes (p<0.001) and sprouts per node (p<0.001) when treated with either GRIN2D siRNA duplex, compared to the scrambled control duplex, suggesting GRIN2D plays some role in the endothelial capillary tube formation (Figure 2A–2C).

The rate of endothelial cell migration is an important indicator of functionality. To determine the effect GRIN2D knockdown has on endothelial migration, scratch wound assays were performed. In this assay, scratches are scored through a confluent monolayer of endothelial cells. Three scratches are scored from top to bottom and three from left to right, generating nine intersecting sites into which endothelial cells will migrate, closing the scratch. By determining the percentage of the scratch wound still open at the point where the control duplex is 90% closed, the rate of endothelial migration can be compared. The percentage scratch wound closure was significantly reduced by GRIN2D knockdown by both duplexes, when compared to the scrambled control (p<0.001), suggesting that GRIN2D also plays a significant role in endothelial migration (Figure 2D&2E).

Endothelial migration takes many forms, and the scratch wound assay cannot accurately assess this alone. The transfilter assay tests the ability of the endothelial cell to transmigrate through a filter, stimulated by a growth factor and mitogen concentration gradient. In order to assess the effect the loss of GRIN2D has on transmigration, this assay was performed. The experimental set up is outlined in Figure 2F, and is summarised in the methods section.

The percentage of cells that have migrated across the membrane was significantly reduced by GRIN2D knockdown by both duplexes when compared to the scrambled control (p<0.001), suggesting that GRIN2D plays a role in endothelial transmigration (Figure 2G).

### Designing a GRIN2D-based tumour vaccine

As the loss of GRIN2D was found to have anti-angiogenic effects *in vitro*, it was investigated as a potential target for anti-angiogenic vaccination. To stimulate host immune response against endogenous GRIN2D, a unique, extracellular epitope was identified. GRIN2D is an ionotropic NMDA receptor subunit, and as such has been predicted to have an intracellular C-terminus, and an extracellular N-terminus [16]. Between these termini, there are predicted to be four transmembrane regions, the second of which forms the ion channel in conjunction with three other subunits. This has been described as a putative structure for the NR2 subunits, with analogous structures in the AMPA glutamate receptor confirmed by fluorescence-detection size-exclusion chromatography [17]. The FASTA sequence of murine GRIN2D (mGRIN2D) was aligned with all other GRIN2 subgroup members using CLUSTAL Omega software (EMBL-EBI, Cambridge, UK), and areas of unique sequence cross-referenced with predicted extracellular regions. A sequence of 78 amino acids unique to mGRIN2D was identified (Supplementary Figure S2), and underwent comparative sequence analysis using the Protein Basic Local Alignment Search Tool (BLASTp) tool (NCBI, Bethesda, MD, USA) to ensure there was no significant sequence homology with any other murine protein, thereby decreasing the likelihood of off-target effects of any future targeted therapy.

The selected GRIN2D peptide sequence was generated as a fusion protein with the Fc fragment of human IgG1 using the pIgG vector expression system in HEK293T-cells. The Fc tag enables interaction with the Fc receptors of circulating immune cells, thereby increasing the immune response against the fusion partner [18], in this case GRIN2D. The resultant GRIN2D-Fc protein was affinity purified using a protein A column. Protein purity was confirmed by SDS-page gel electrophoresis (Supplementary Figure S2).

### Vaccination against GRIN2D impairs angiogenesis and tumour growth

**Figure 3:**
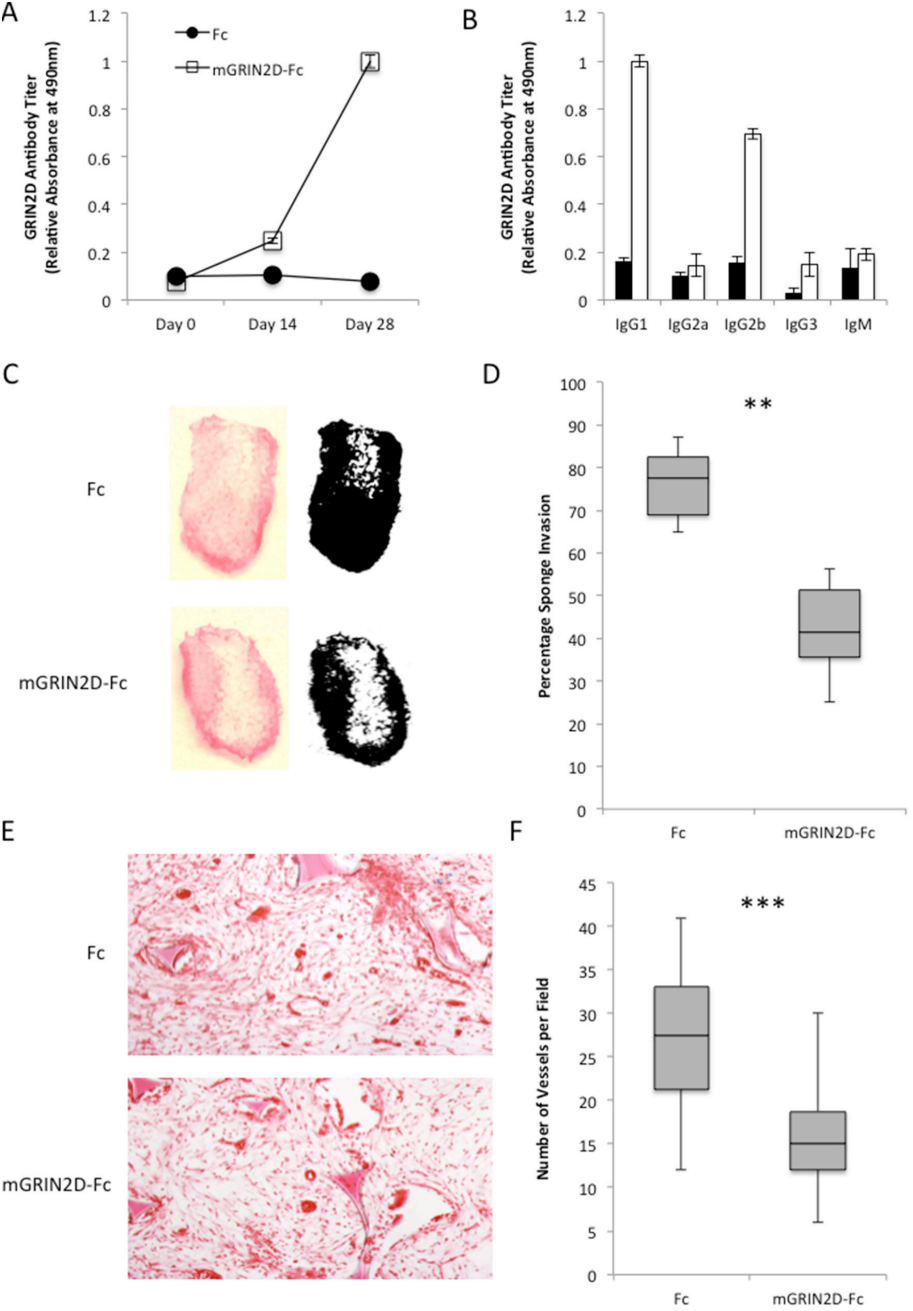
The physiological effects of GRIN2D-Fc vaccination **A-B.** quantitation of immune response to vaccination with GRIN2D-Fc fusion protein by ELISA, showing A, overall response and B, IgG specific response. Vaccinated mice had sponges introduced into their flank and angiogenesis was stimulated into the sponge with FGF infusions. **C.** representative images of macroscopic vascular invasion into the sponge in the treated and untreated groups. A mask was generated in image J [54] for each sponge and the percentage sponge invasion quantified, **D, E.** representative H&E images of subcutaneous sponge morphology. **F.** quantitation from the H&E images of sponge vessel density. Statistical analysis for all, Mann-Whitney U, ***P<0.0001, **P<0.001. 6 sponges in each group, 5 microscopic fields per sponge.

To investigate the *in vivo* effects of targeting GRIN2D by vaccination, mGRIN2D-Fc fusion protein was administered in emulsion with Freund's adjuvant to separate groups of six mice, with Fc alone in Freund's administered to the control group. Utilising this approach, mice received 50 μg of mGRIN2D-Fc fusion protein subcutaneously with 100 μl of Freund's adjuvant at day 0 and day 14, with a terminal cardiac bleed taken at day 28 (Supplementary Figure S3). ELISA was utilised to quantify the specific immune response to GRIN2D, and to differentiate the immunoglobulin isotype involved in the response (Figure 3A&3B). This analysis confirmed a specific immune response to GRIN2D, predominated by IgG1 and IgG2b, indicating a Th-2 T-cell response, consistent with immunisation [19]. Importantly, there was no evidence of a significant difference between groups in antibody response to the Fc component of the vaccine, when measured by ELISA (p=0.379, Mann Whitney).

The effects of targeting GRIN2D on *in vivo* angiogenesis was investigated using the subcutaneous sponge assay. Following vaccination with mGRIN2D-Fc, sponges were paraffin embedded, sectioned and stained with haematoxylin and eosin (H&E). Random fields were assessed for blood vessel count. A significant decrease in both sponge invasion (p=0.0026) and vessel density (p<0.0001) was observed compared to controls (Figure 3C–3F), implicating GRIN2D in the process of angiogenesis *in vivo*.

**Figure 4:**
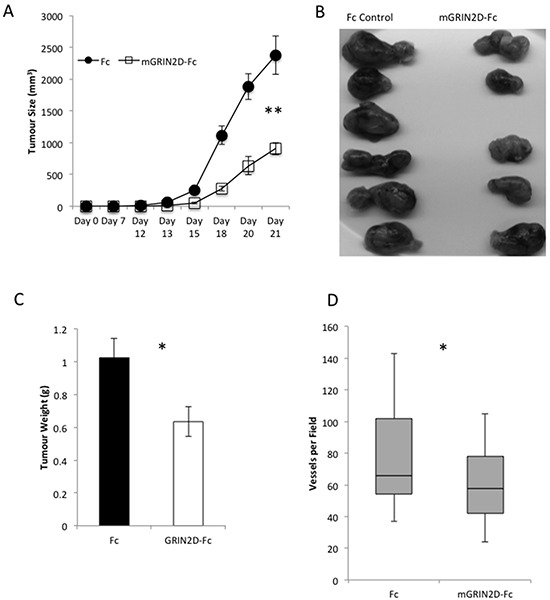
GRIN2D-Fc vaccination decreases subcutaneous CT26 tumour growth and vascularity CT26 tumour cells were inoculated into vaccinated and non-vaccinated Balb/c mice. **A.** tumour growth was monitored by caliper measurement. Tumours were excised at day 21, imaged, **B.** and weighed, **C, D.** representative H&E images of subcutaneous tumour morphology. **E.** quantitation from the H&E images of tumour vessel density. Statistical analysis for all, Mann-Whitney U, ***P<0.0001, **P<0.001.5 mice per group; 5 microscopic fields per tumour.

This vaccination approach was extended to target tumour vessels and angiogenesis in a murine model of CRC (CT26). Tumours grown in mice vaccinated against GRIN2D were found to display a significant decrease in tumour growth by calliper measurement (p=0.004, Mann-Whitney) (Figure 4A). This was corroborated by analysis of excised tumour appearance and weight (p=0.0274) (Figure 4B&4C). Interestingly, RTqPCR quantification of GRIN2D expression within the endothelial compartment of the excised tumours endothelial cells demonstrated a 5.5-fold upregulation of expression within the mGRIN2D-Fc treated group compared to controls, when adjusted for endothelial isolation efficiency (p=0.0069, Mann-Whitney). Vascular density was quantified in H&E sections of excised CT26-Luc tumours, indicating a significant decrease in the numbers of vessels per field in five random microscopic fields per tumour (mean 78 vs. 62 vessels per field, p=0.0367, Mann Whitney) (Figure 4D). This result demonstrates the utility of GRIN2D vaccination as an anti-angiogenic approach to treat colorectal cancer.

## DISCUSSION

Microarray transcriptomic analysis on freshly isolated endothelium from colorectal cancer and matched healthy colon has identified a panel of putative colorectal tumour endothelial markers. Of these the glutamate receptor GRIN2D emerged as the most interesting target. GRIN2D was shown to have vessel-restricted expression in colorectal cancer by RTqPCR and IHC analysis. Multi-organ tissue array analysis identified GRIN2D to be predominantly expressed in tumour tissue, particularly in colorectal malignancies, where its expression was shown to be associated with increased survival. *In vitro* analysis of GRIN2D's endothelial function showed it to be important in endothelial migration and capillary tube formation in matrigel. *In vivo* analysis showed GRIN2D vaccination to be an effective approach to inhibit both physiological and tumour-associated angiogenesis.

GRIN2D is primarily studied as a neuronal ionotropic glutamate receptor, involved in long-term potentiation, and plays a role in certain types of learning and memory. GRIN2D is a component of the N-methyl-D aspartate (NMDA) receptor calcium channels, which are heterotetramers formed from a combination of GRIN1 and either GRIN2 or GRIN3 subunits [20]. It is widely expressed within the brain, in particular the developing diencephalon, however, selective knockout mice develop normally both pre- and post-natally, with normal mating behaviour [21]. No observed effect is described in motor activity or anxiety tests among selective knockout mice, however, due to the loss of function of certain NMDA receptors, mice showed altered emotional behaviour in forced swimming and light/dark box stress tests [22]. This led to the suggestion of its role in the glutaminergic theory of schizophrenia, interest in which has been a focus of much research, reviewed in Moghaddam and Javitt, 2012 [23].

Neuronal and vascular cells share an embryonic stem cell lineage, leading to considerable cross-expression of endothelial and neuronal genes and many similarities in the processes that govern the modelling of vascular and neuronal architecture [24]. With this in mind it is unsurprising that a traditionally neuronal glutamate receptor GRIN2D should be found to be expressed on endothelial cells.

GRIN2D's identity as a component of a calcium channel could be responsible for its observed role in angiogenesis. VEGF, a potent stimulatory growth factor of angiogenesis, promotes endothelial migration, proliferation and capillary formation in part via VEGF-mediated calcium influx [25]. Increased intracellular calcium concentration, observed or induced by arachidonic acid, has been reported to promote endothelial migration and tube formation in angiogenesis assays [26, 27]. Loss of GRIN2D by siRNA knockdown results in reduced endothelial migration, tube formation and transmigration, which could be in part due to reduced calcium influx into the endothelial cells, curtailing the efficiency of growth factor mediated endothelial function, although this theory requires further investigation.

The functioning of GRIN2D in neuronal calcium channels is distinct from that of other NMDA receptor components, in that it promotes calcium influx over an extended period, mediating its role in long term potentiation [28, 29]. It is therefore easy to see how, by offering long-term calcium influx, the promotion of GRIN2D containing calcium channels in tumour endothelium could offer a survival advantage and even prime endothelial cells to respond to pro-angiogenic growth factor signalling within the tumour. Calcium flux and metabolism has been suggested as a target for anti-angiogenic therapy [30] and targeted inhibition of GRIN2D could be a potential method for achieving this.

Multiple links have been described between glutamate receptor function and tumour biology, with knockdown of some non-GRIN2D glutamate receptor subunits resulting in both pro and anti-tumour effects [31]. Additionally increased serum levels of glutamate have been reported as a marker of malignant potential in prostate cancer [32], where it has also been observed to be positively related to advancing Gleason grade [33]. It has been proposed that this is due to glutamate acting to cause a genetic switch within the tumour, reducing the threshold for oncogenic k-ras signalling [34]. This is of particular relevance in colorectal cancer as mutations in the k-ras oncogene is strongly implicated in the progression of colorectal cancer [35].

GRIN2D shows a promising tumour specific endothelial expression profile in a number of tumours, in particular colorectal cancer. However, it also appears to be expressed in a small subset of healthy tissues of the colon, stomach, esophagus and larynx as well as its reported expression throughout the brain and autonomic and enteric nervous systems [36]. The integrity of the blood brain barrier (BBB) and other nervous system protective structures must be ensured and maintained to avoid potentially serious side effects from GRIN2D guided therapies. If the BBB is intact the safety of immunological therapies should still be maintained, as large proteins like antibodies, cannot pass through the tight endothelial junctions of the BBB [37]. However, in 2-3% of cases of advanced CRC, patients develop brain metastases leading to disruption of the BBB [38]. This could make targeting GRIN2D potentially unwise in this setting. With this in mind, care will have to be taken in the selection of patients in which GRIN2D targeted therapies can be safely used.

Intriguingly GRIN2D expression in the vessels of colorectal cancer appears to be strongly associated, albeit not significantly so, with improved patient survival. The expression of tumour endothelial markers being associated with survival in this way is not without precedent. Expression of the tumour specific orphan receptor ELTD1 has been reported to be associated with improved survival in head and neck, colorectal and ovarian cancers [39]. Increased expression of ROBO4, CLEC14A and ECSCR have also been associated with improvements in survival in non-small cell lung cancer [40]. It has been suggested that the expression of these pro-angiogenic TEMs could be associated with vascular normalisation within tumours. Indeed ROBO4-Slit2 signalling has been implicated in the normalisation of VEGF induced chaotic vascular structures [41]. This vascular normalisation could in turn lead to improved perfusion of the tumours leading to greater responsiveness to systematic blood-borne chemotherapeutics and therefore increased survival [39, 40].

Immune therapy is an established, yet still rapidly expanding field within anti-cancer therapy, with monoclonal antibody therapy forming the bulk of approved immune therapies in colorectal cancer. The field is constantly evolving, and novel applications have been described, including the use of a photosensitiser conjugated to an antibody specific to the Extra Domain B (EDB) of fibronectin, which demonstrated complete tumour ablation in a murine xenograft model of embryological tumour growth [42]. Other groups have focused on the generation of chimeric antigen receptor regulatory T-cells to selectively target the vasculature, for instance through the targeting of VEGFR2 in multiple murine cell lines, including the CT26 line [43]. Work has been performed to this end in colitis-associated colorectal cancer using CEA-specific Chimeric Antigen Receptor (CAR) regulatory T-cells [44].

Vaccination-based targeting of endothelial cell function, demonstrated for GRIN2D in this paper, offers certain benefits over these other novel approaches to tumour targeting, not least its potential for a relative lack of cost, due to the persistence of the immune response, and the avoidance of regular drug infusions. Having said this, research has shown that vaccination against a self-antigen does not result in a persistent immune response, with circulating antibodies returning to baseline after seven months [45]. It is also important to note that for vaccination to be a successful strategy, it requires the host to possess an intact immune system. This is often compromised in patients with malignancy, either from their malignancy itself [46], or from the effects of exogenous steroids [47], chemotherapeutic agents [48], opioids [49] and even the emotional stress brought on by a cancer diagnosis and treatment [50]. Mechanisms are highly varied, but include T-cell mediated immunity, which may impact on the potential success of a pure vaccine-based treatment in this context.

In conclusion, GRIN2D is a promising vascular target in colorectal cancer for both treatment and prognostication. Endothelial function is impaired by siRNA-mediated disruption of gene function in *in vitro* angiogenesis assays and vascularisation is inhibited in *in vivo* models of normal and tumour angiogenesis. This effect may be due to reduced calcium flux within the endothelial cells, although this theory requires further investigation. The expression profile of GRIN2D throughout the body must be further assessed in order to ascertain the safety of systemic administration of GRIN2D targeted therapies.

## MATERIALS AND METHODS

### Quantitative reverse transcription polymerase chain reaction (RTqPCR)

RNA isolation and cDNA production was performed as previously described [51]. RTqPCR was performed using the Exiqon universal probe system (Roche, Burgess Hill, UK) as previously described [52]. Gene expression of samples was assessed in triplicate using the delta-delta CT method normalised to flotillin-2 unless otherwise stated. Primer sequences follow:

**Table:** 

PECAM-1	Human	F: 5′-GCAACACAGTCCAGATAGTCGT-3′ R: 5′-GACCTCAAACTGGGCATCAT-3′
Flotilin-2	Human	F: 5′-GATCCTCAGCTTCACCATCAA-3′ R: 5′-TCAGCATCTCTCTGCACCAC-3′
GRIN2D	Human	F: 5′-GGCTCAGTGACCGCAAGT-3′ R: 5′-GCACGGTCCCAAACTTCA-3′

### Immunohistochemical staining

Paraffin embedded sections were initially placed in xylene for 5 mins, then into 100% ethanol for 5 mins, then rehydrated by washing in water. Endogenous peroxidase block was performed using 0.3% hydrogen peroxide for 15 mins. The slides were then washed with water, and antigen retrieval was performed by incubating overnight in 1 mM EDTA pH 8.0 with 0.1% Tween in a beaker at 65°C, with stirring overnight. The following day, the beaker was cooled with running water until at room temperature. The slides were then washed with PBS for 5 mins, and then were treated with 100 μl of 2x casein block. The slides were then incubated with 2 μg/ml mouse polyclonal antisera to CD31 (clone JC70, Dako, Ely, UK) or a custom antibody raised against the extracellular region of GRIN2D (Eurogentec, Southampton, UK) for 1 hour. The sections were then stained and visualized using the ImmPRESS universal antibody kit and ImmPACT NovaRed chromagen (Vector labs, Burlingame, CA, USA). The sections were counterstained with Mayer's hematoxylin (Sigma), dehydrated and mounted in distyrene–plasticizer–xylene resin (Sigma). All images were acquired using a Leica DM6000 light microscope (Leica, Milton Keynes, UK). The following tissue arrays were used: MA2, MAN2, MC4, MCN4, CD4, CDN4, CDA3 (Superbiochip, Seoul, Korea).

### siRNA knockdown of novel TEMs in HUVEC

Human umbilical cord vein endothelial cells (HUVEC) were isolated from human umbilical cords as previously described [53] and used at passage 2. 1.75×10^5^ HUVEC were plated into each well of a 6-well plate coated in 0.1% gelatine. These cells were incubated overnight at 37°C. A mix of 2.5 μl of 20 μM siRNA duplex and 167.5 μl Optimem (final duplex concentration of 50 nM), and a mix of 3 μl RNAiMAX lipofectamine and 27 μl Optimem were incubated at room temperature for 10 mins. 30 μl of lipofectamine mix was added to the duplex mix, flicked to combine, and incubated at RT for 10 mins. The seeded cells were then washed twice with PBS, and 800 μl of Optimem was added. Then 200 μl of lipofectamine/duplex mix was added to the cells, and incubated at 37°C for 4 hrs. At this stage, the media was aspirated off, and replaced with cM199 without antibiotics. The following day, the cells were harvested and assays performed.

### Western blot

HUVEC were harvested by scraping, lysed, and subjected to 8% SDS-polyacrylamide gel electrophoresis. The protein was blotted onto nitrocellulose, stained with 0.3 μg/ml rabbit polyclonal antisera to MCAM (HPA008848, Atlas Antibodies, Sigma), visualized with ECL peroxidase linked donkey anti–rabbit IgG (NA9340V, GE Healthcare) and ECL detection reagent (GE Healthcare) and used to develop Amersham Hyperfilm™ ECL (GE Healthcare).

### Scratch wound assay

3×10^5^ HUVEC were plated into each well of a 6-well plate and cultured in cDMEM at 37°C. Once the cells were confluent, a P200 pipette tip was used to make three parallel scratches both vertically and horizontally, resulting in 9 intersections. The plate was then washed twice with PBS to remove any floating cells. Images were taken at 0 hrs, 6-8 hrs and 16-24 hrs to measure wound closure.

### Matrigel tube forming assay

A 1 ml aliquot of Matrigel was retrieved from the −80°C freezer, and allowed to defrost overnight in the cold room at 4°C and on ice. The following day, the wells of a 12-well plate were moistened with PBS, which was then removed to leave a thin film within the well. 70 μl of Matrigel was added to each well, and then allowed to solidify at 37°C for 30 mins. 1.4×10^5^ cells were added to each well in 1 ml cM199. The assay was incubated for 16-24 hrs, and then images acquired to measure tube number, length and branching.

### Transwell filter assay (modified boyden chamber assay)

700 μl of 0.1% gelatine was added to each well of a 24-well plate, and then a cell permeable membrane was placed into the well, followed by a further 200 μl of gelatine. This was incubated at 37°C for 10 mins, then aspirated off. 700 μl of cM199 was added to the bottom of the well, with 3×10^4^ HUVEC in low-serum M199 (1% FCS & no brain extract) placed on top of the filter. The assay was then incubated for 16-24 hrs.

Following incubation, the media was removed, and the wells washed with 700/200 μl of PBS. The PBS was then removed and replaced with equivalent volumes of fixing solution containing 2% Formaldehyde in PBS + 2 μg/ml bisbenzamide for 15 mins. The fixing solution was then removed and the wells and filters washed with PBS.

To mount the filter, initially a drop of PBS was placed onto a slide, and then the filter was placed on top of this. A scalpel was used to excise the filter from its mounting, and the excess PBS wiped away. A drop of Shandon Immu-Mount(TM was added to the filter, and a coverslip applied. The slides were then stored in the dark at 4°C until ready to be imaged. To quantify this assay, the slide was visualised at 20x magnification, and counts made of those cells atop and below the filter by adjustment of the focus. These scores from 5-10 sites on the slide were averaged, and the figures used to quantify percentage transmigration.

### Generation of vector containing GRIN2D-GST fusion DNA

The insert DNA for the extracellular domain of GRIN2D was digested out of the GRIN2D-PIgG vector using the *Eco*R1 and *Bam*H1 restriction sites using a double digestion, and ligated in a single reaction into the pGEX-2T vector (GE Healthcare, Life Sciences, Little Chalfont, UK) using the same sites and T4 ligase. Following ligation, heat-shock transformation of ‘gold efficiency α-select' cells was performed, followed by overnight culture on ampicillin plates. Colonies were picked and grown up overnight, and then DNA extracted using the GeneJET Miniprep kit. These samples were sequence verified using the forward and reverse pGEX sequencing primers (GE Healthcare, Life Sciences, Little Chalfont, UK). The derived construct was scaled up by maxi-prep and stored at −20°C. This vector was named the pGEX2T-GRIN2D Vector.

### Polyethylenimine (PEI) transfection of HEK293T cells

For a 15 cm plate, on the day before transfection 6×10^6^ HEK293T cells were plated out in 20 ml cDMEM. The following day, 18 μg of DNA was combined with 2 ml of Optimem serum-free media, and mixed by flicking of the tube. 72 μg of PEI was then added and briefly vortexed at low speed to mix, followed by a 10 min incubation at room temperature. The mixture was then gently added to the HEK293T cells and mixed by north/south east/west movements.

### Murine subcutaneous colorectal cancer model

Mice were handled and treated in accordance with home office requirements (Licence number, PPL. 40/3339). Balb/c mice were anaesthetised using inhaled Isofluorane, and following confirmation of depth of anaesthesia with a toe pinch, the left flank was shaved. Anaesthesia was maintained using a facemask. 1×10^5^ Luciferase-transduced CT26 (CT26-Luc) colorectal cancer cells were injected into the left flank of each mouse. A one-week tumour establishment period was allowed, following which regular tumour calliper measurement was performed. This was combined with intraperitoneal injection of 200 μl of 15 mg/ml D-Luciferin in PBS, and tumour growth measurement using the intravital imaging system (IVIS).

### Murine subcutaneous sponge model

Buprenorphine was administered 1 hour prior to surgery. NSAIDs were avoided due to potential confounding effects on angiogenesis. Balb/c mice were anaesthetised using inhaled Isofluorane, and following confirmation of depth of anaesthesia with a toe pinch, both flanks were shaved. The skin was cleansed with ethanol. A small incision was made in the flank and a subcutaneous pocket created by blunt dissection. A 0.5 × 1.0 cm cylindrical sponge was inserted into the sub-cutaneous pocket and the skin closed using 6/0 absorbable subcuticular sutures. This was then repeated for the other flank. Over the next 14 days, 100 μl of 20 ng/ml bFGF was injected into the sponge a total of 7 times, with a minimum of 12 hrs between injections. Animals were culled by a schedule 1 method, and the sponges fixed in 4% formalin prior to histological analysis.

### Murine vaccination model

Balb/c mice were anaesthetised using inhaled Isofluorane, and following confirmation of depth of anaesthesia with a toe pinch, 100μl Freund's Complete Adjuvant was injected subcutaneously with 50μg of either treatment or control protein. After 14 days, 100μl Freund's Incomplete Adjuvant was injected subcutaneously with 50μg of either treatment or control protein to boost the host antibody response. Tail vein bleeds were performed at days 0, 14 and 28, with antibody response quantified by ELISA.

### Enzyme-Linked immunosorbent assays (ELISA)

A Nunc Maxi-Sorp™ ELISA plate (eBioscience, Hatfield, UK) was coated with 100 μl of 2 μg/ml of GRIN2D-GST or Fc protein, and incubated overnight at 4°C. The plate was blocked using 200 μl 3% BSA in PBS-T for at least 2 hrs. The plate was then washed 5 times with 100 μl PBS-T, and then 5 μl of serum in 45 μl PBS was incubated overnight in each well at 4°C. The following day, the serum was removed, and the plate washed 5 times. For antibody isotyping, 100 μl of primary antibodies at 1 in 500 were incubated for 90 mins. The plate was then washed 5 times. Peroxidase-conjugated anti-mouse IgG (Dako, Ely, UK) was then applied as a secondary for 90 mins. For Ig Isotyping, IgG1, IgG2a, IgG2b, IgG3, IgM antibody (Sigma, Gillingham, UK) was applied as a secondary, then visualised using rabbit anti-goat-HRP antibody (Dako, Ely, UK). The plate was incubated for 15 mins in detection solution (10 mg o-phenylenediamine dihydrochloride (OPD) in sodium perborate/phosphate citrate buffer), and then quenched with 50 μl 3M HCl. Imaging was performed using a plate reader at 490 nm.

### Vascular quantification

Following excision and formalin fixing of either sponges or tumours, specimens were paraffin embedded and mounted as 10μm sections. These then underwent staining with haematoxylin and eosin, and blood vessels counted in 5 random fields per sample at 10x magnification.

## SUPPLEMENTARY FIGURES AND TABLES


