# Supplementary material for: Glutamate dependent NMDA receptor 2D is a novel angiogenic tumour endothelial marker in colorectal cancer
Source: Oncotarget. 2016 Mar 1;7(15):20440–54. doi: 10.18632/oncotarget.7812 (PMC4991466; doi:10.18632/oncotarget.7812)
Supplement: Supplementary file 1 [file oncotarget-07-20440-s001.pdf]

SUPPLEMENTARY FIGURES AND TABLES

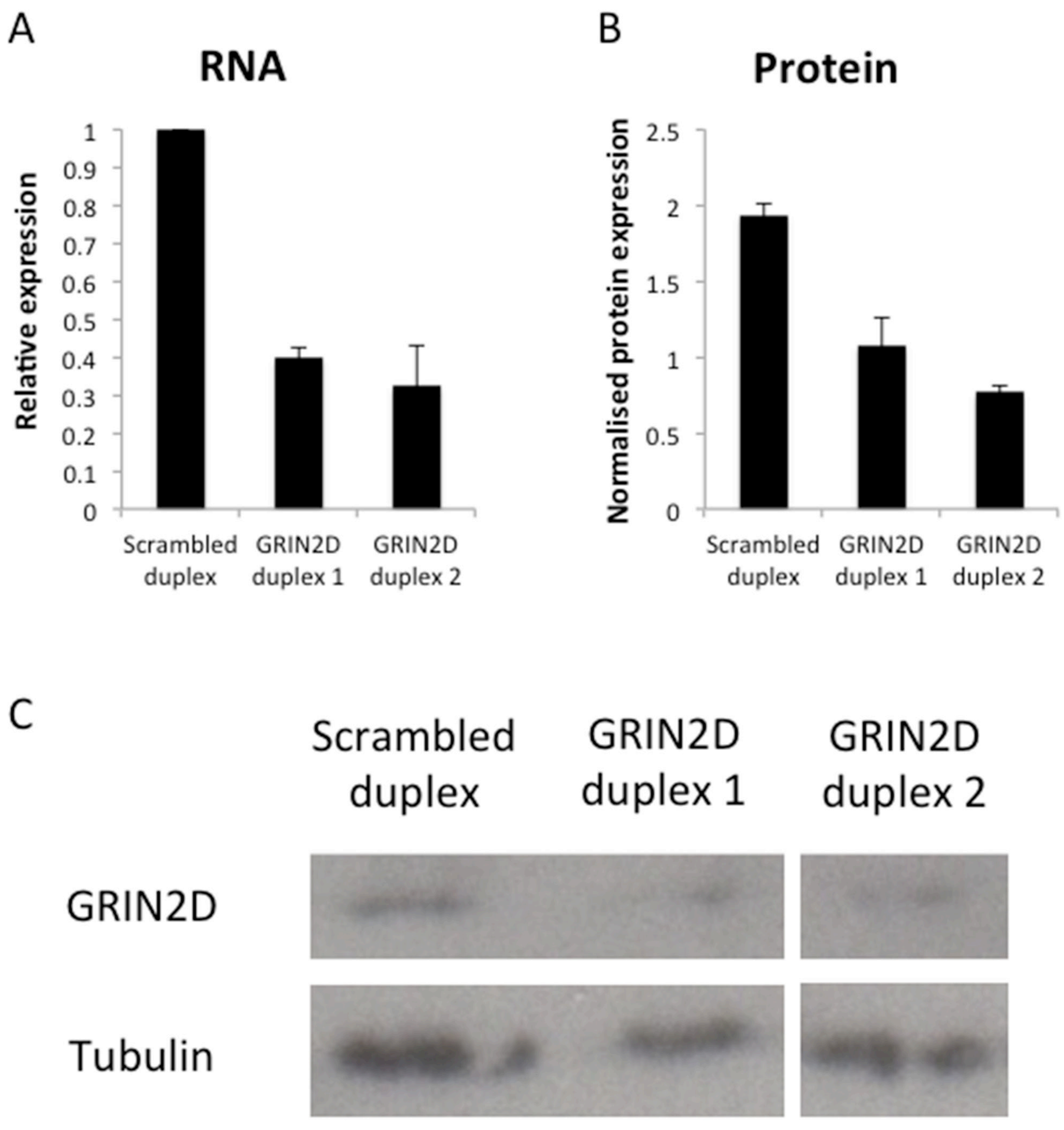

Supplementary Figure S1: siRNA duplexes successfully knockdown GRIN2D expression on the RNA and protein level. Analysis of GRIN2D expression in three cords of HUVEC treated with a scrambled duplex or two GRIN2D targeting duplexes, by **A.** RTqPCR (normalised to flotillin-2) or **B.** western blot (normalised to Tubulin) (n=3, average expression ± SEM). **C.** representative western blot images (GRIN2D band - 70 kDa, Tubulin band – 55 kDa).

A

```
                1                                                          60
GRIN2D      MRGAGGPRGPRGPAKMLLLLALACASPFPEEVPGPGAAGGGTGGARPLNVALVFS--GPA
GRIN2A      ----------MGRLGYWTLLVLPALL----VWHGPAQNAAAEKGTPALNIAVLLG-HSHD
GRIN2B      ----MKPSAECCSPKFWLVLAVLAV---------SGSKARSQKSAPSIGIAVILVGTSDE
GRIN2C      ---MGGA---LGPALLLTSL------------LGAWAGLGAGQGEQAVTVAVVFGSSGPL
                                  78
GRIN2D      YAAEAARLGPAVAAAVRSPG
GRIN2A      VTERE-LRNLWGPEQATGLP
GRIN2B      VAI----KDAHEKDDFHHLS
GRIN2C      QAQARTRLTPQ---NFLDLP
```

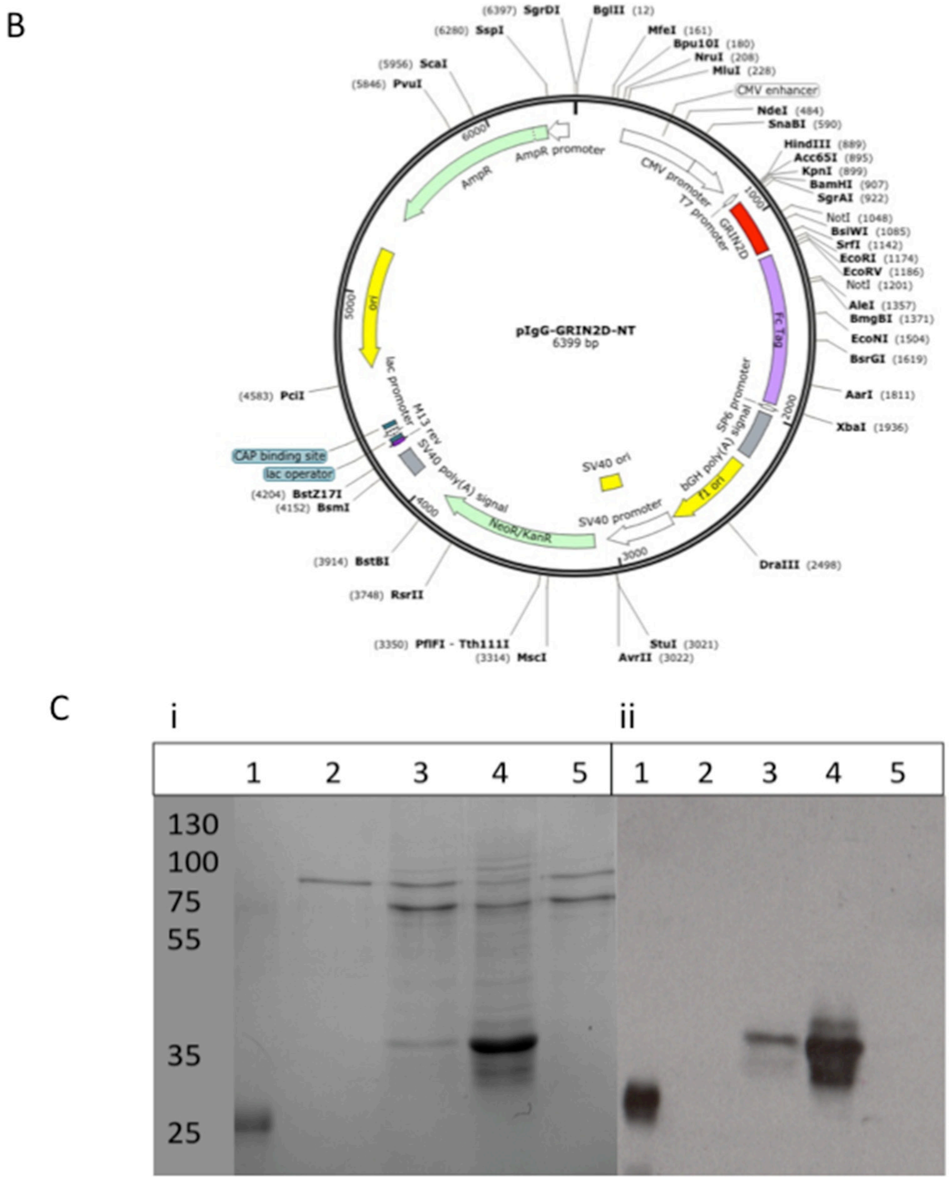

Supplemental Figure S2: Construction and production of GRIN2D-Fc fusion protein. A. The N-terminal sequence (bp 1-78) was identified as unique and specific to GRIN2D. **B.** This peptide was ligated into the pIgG (pcDNA3.1) vector for onward production in HEK-293T cells. Secreted media was collected and affinity purified using a protein A column. **C.** Corresponding Coomassie Stain (i) and anti-Fc Western Blot (ii) of 12% SDS-PAGE Gel. Lane 1 – Purified Human Fc. Lane 2 – Optimem. Lane 3 – Secreted Media. Lane 4 – Purified GRIN2D-Fc. Lane 5 – Depleted Media. Predicted size of hGRINDFc = 34 kDa; Fc = 26 kDa.

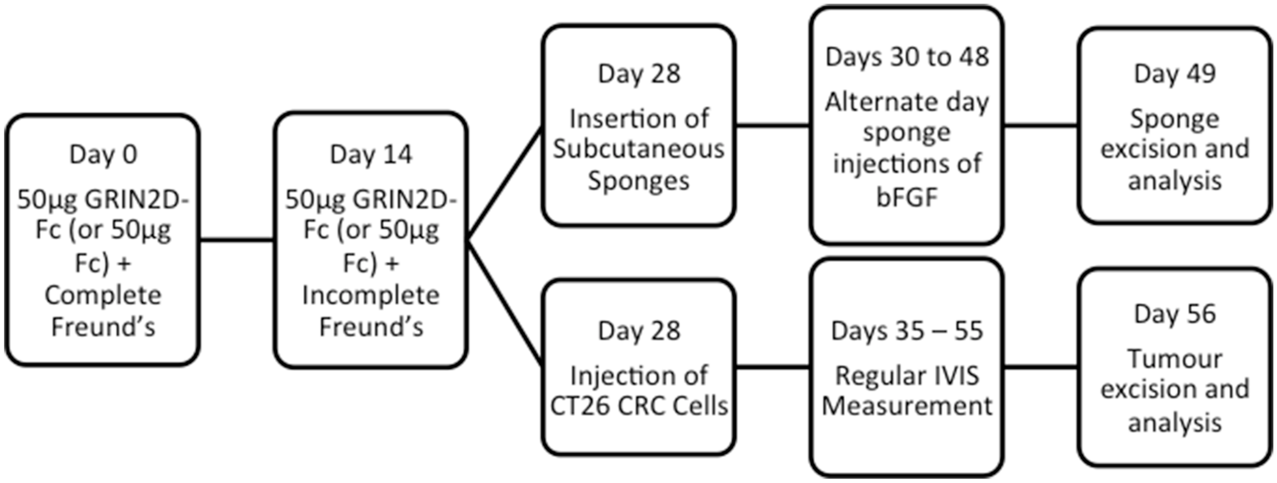

Supplementary Figure S3: Protocols for murine vaccination model followed by either subcutaneous sponge or subcutaneous CT26-luc tumour implantation.

Supplementary Table S1: Matrix metallopeptidases enriched in colorectal cancer vessels

Gene ID	Gene Symbol	GeneBank accession no.	LogFC	P-value
matrix metallopeptidase 3 (stromelysin 1, progelatinase)	MMP3	NM_002422	5.72	0.01
matrix metallopeptidase 7 (matrilysin, uterine)	MMP7	NM_002423	5.54	0.00
matrix metallopeptidase 12 (macrophage elastase)	MMP12	NM_002426	4.86	0.00
matrix metallopeptidase 1 (interstitial collagenase)	MMP1	NM_002421	4.49	0.04
matrix metallopeptidase 9 (gelatinase B)	MMP9	NM_004994	4.10	0.00
matrix metallopeptidase 11 (stromelysin 3)	MMP11	NM_005940	3.25	0.01
matrix metallopeptidase 10 (stromelysin 2)	MMP10	NM_002425	2.54	0.10
matrix metallopeptidase 14 (membrane-inserted)	MMP14	NM_004995	1.94	0.04
matrix metallopeptidase 2 (gelatinase A)	MMP2	NM_004530	1.51	0.16
matrix metallopeptidase 13 (collagenase 3)	MMP13	NM_002427	1.18	0.11

Supplementary Table S2: Collagens enriched in colorectal cancer vessels

Gene ID	Gene Symbol	GeneBank accession no.	LogFC	P-value
collagen, type I, alpha 1	COL1A1	NM_000088	4.18	0.00
collagen, type XII, alpha 1	COL12A1	NM_004370	3.13	0.00
collagen, type IV, alpha 1	COL4A1	NM_001845	2.86	0.00
collagen, type I, alpha 2	COL1A2	NM_000089	2.79	0.01
collagen, type XV, alpha 1	COL15A1	NM_001855	2.56	0.01
collagen, type XVIII, alpha 1	COL18A1	NM_030582	2.42	0.00
collagen, type IV, alpha 2	COL4A2	NM_001846	2.36	0.00
collagen, type V, alpha 1	COL5A1	NM_000093	2.30	0.04
collagen, type VIII, alpha 1	COL8A1	NM_001850	2.28	0.02
collagen, type V, alpha 2	COL5A2	NM_000393	2.10	0.03
collagen, type VII, alpha 1	COL7A1	NM_000094	2.00	0.17
collagen, type V, alpha 3	COL5A3	NM_015719	1.79	0.01
collagen, type X, alpha 1	COL10A1	NM_000493	1.69	0.06
collagen, type III, alpha 1	COL3A1	NM_000090	1.66	0.10
collagen, type XIV, alpha 1	COL14A1	NM_021110	1.54	0.08
collagen, type VI, alpha 1	COL6A1	NM_001848	1.45	0.14
collagen, type VI, alpha 3	COL6A3	NM_004369	1.44	0.08
collagen, type IX, alpha 1	COL9A1	NM_001851	1.08	0.11

Supplementary Table S3: Key clinical data for CRC cohort showing overall survival (OS)

Characteristics	N of patients	Median OS	Mean OS
All	90	87.5	112.8
Gender			
Male	65	86	107.2
Female	25	122	127.3
Age			
above 58	47	122	130.2
below or equal to 58	43	67	93.8
Stage			
I/II	44	157.5	148.5
III/IV	46	38	78.6
Tumour location			
Ascending colon	21	27	63.5
Descending colon	6	194	183
Sigmoid colon	31	84	96.6
Transverse colon	9	186	127.1
Cecum	4	102.5	109
Rectum	19	186	165.6
T-stage			
T2	8	187.5	160.5
T3	75	86	105.5
T4	7	122	136.3
N-stage			
N0	50	129.5	138.3
N1+	40	33	80.1
M-stage			
M0	75	116	126.3
M1	15	42	45.3
GRIN2D staining			
Positive	21	154	137.6
Negative	69	86	109.8
